# Supplementary material for: Atomic-thick metastable phase RhMo nanosheets for hydrogen oxidation catalysis
Source: Nat Commun. 2023 Mar 30;14:1761. doi: 10.1038/s41467-023-37406-y (PMC10063647; doi:10.1038/s41467-023-37406-y)
Supplement: Supplementary file 1 — Supplementary information [file 41467_2023_37406_MOESM1_ESM.pdf]

Supplementary Figures

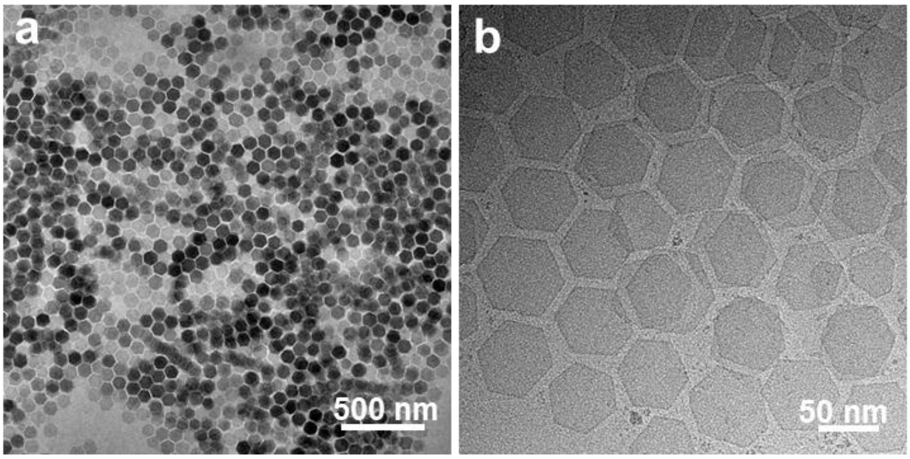

Supplementary Fig. 1 (a, b) TEM images of RhMo NSs.

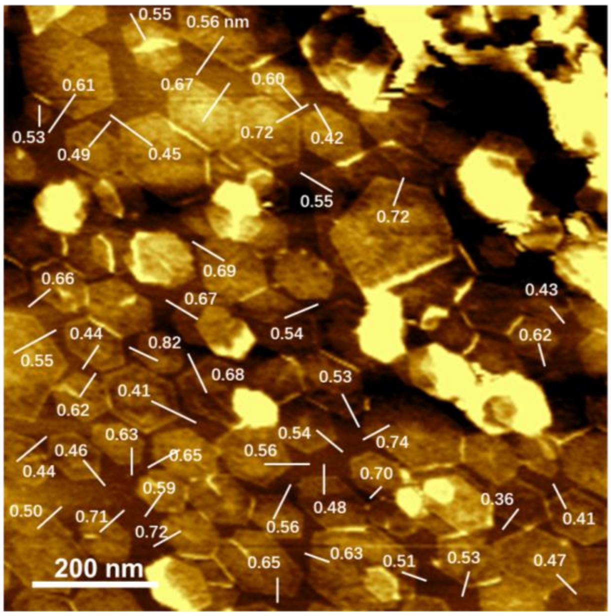

Supplementary Fig. 2 AFM image of RhMo NSs. The bright edge region and the dark core areas indicate RhMo NSs have a typical core/shell structure. Meanwhile, the height profiles (remarked in **Supplementary Fig. 2**) reveal that the thickness of RhMo NSs is around 0.57 nm.

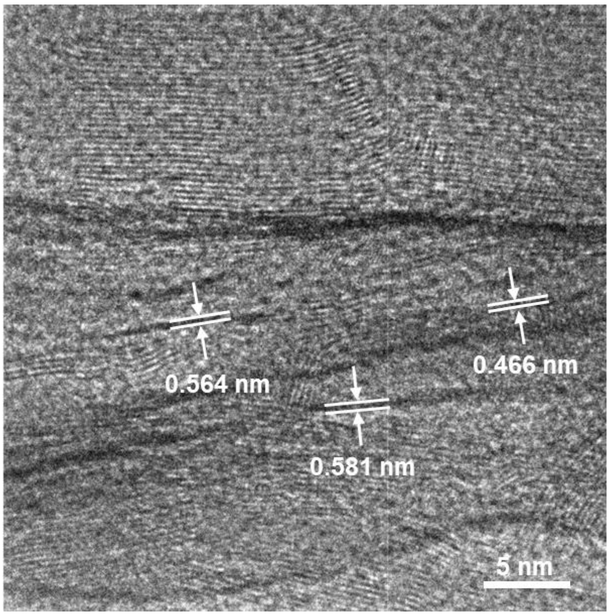

Supplementary Fig. 3 HRTEM image of RhMo NSs loading on carbon nanotubes. A vertical view of RhMo NSs reveals that the thickness of RhMo NSs is around 0.54 nm, being consistent with the AFM result.

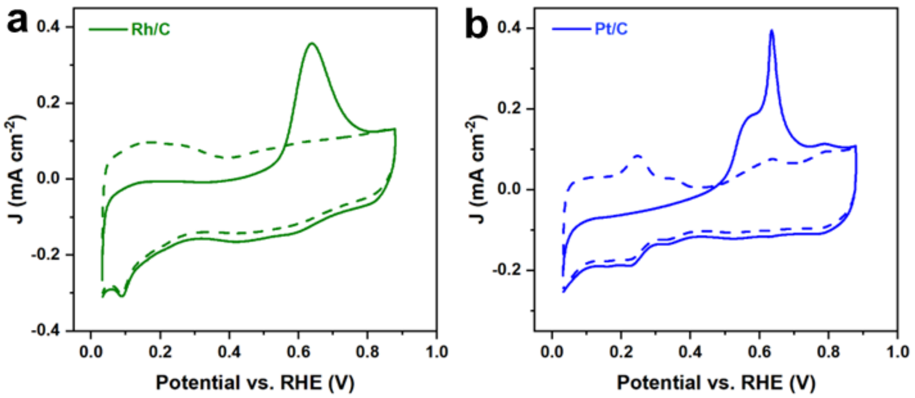

Supplementary Fig. 4 CO stripping voltammograms of (a) Rh/C, and (b) Pt/C. The voltammograms were conducted in 0.1 M KOH. Scan rate, 10 mV s^{-1}.

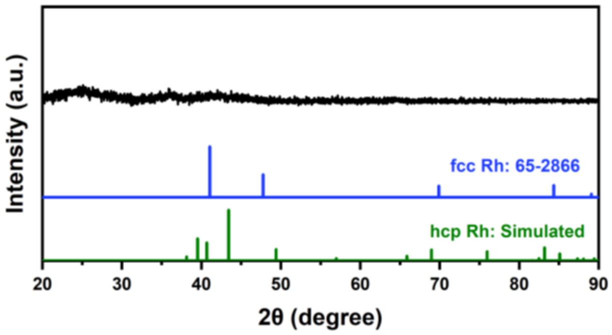

Supplementary Fig. 5 XRD pattern of RhMo NSs.

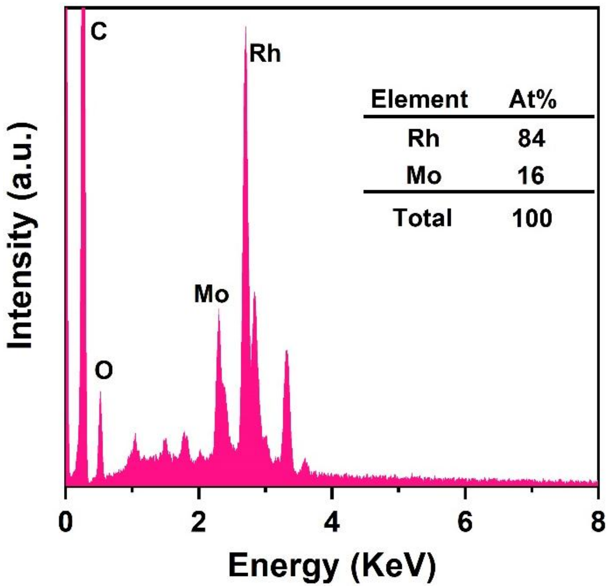

Supplementary Fig. 6 SEM-EDS analysis of RhMo NSs.

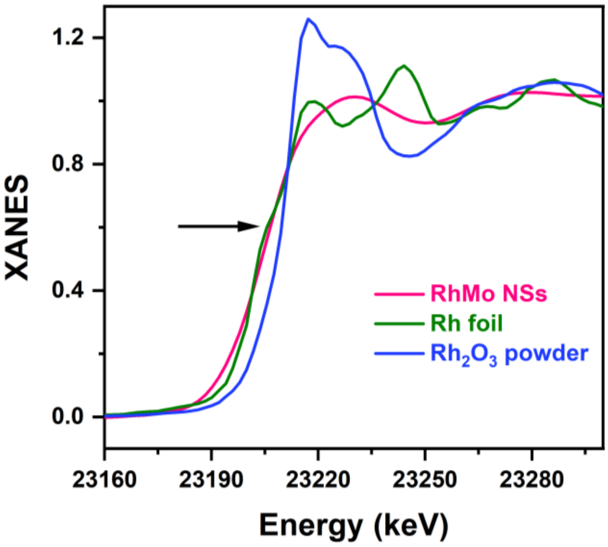

Supplementary Fig. 7 Normalized Rh K-edge X-ray absorption near-edge spectra (XANES) of RhMo NSs, Rh foil, and Rh_2O_3 powder.

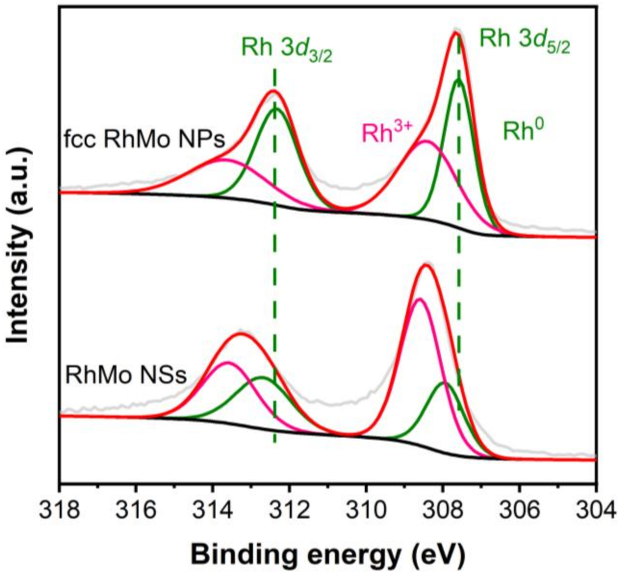

Supplementary Fig. 8 Rh 3*d* XPS spectra of fcc RhMo NPs and RhMo NSs. The main characteristic peaks of Rh^0 can be observed in RhMo NSs, suggesting the metallic state of Rh in RhMo NSs, be consistent with the XANES results. In addition, the peak position of Rh^0 in the XPS spectra of RhMo NSs positively shifts to higher binding energy, suggesting that the surface valence state of Rh in RhMo NSs is higher than that of fcc RhMo NPs. Meanwhile, the ratio of Rh^{3+}/Rh^0 of RhMo NSs is 0.31, which is higher than that of fcc RhMo NPs (0.28), indicating that the ultrathin structure is easy to be oxidized.

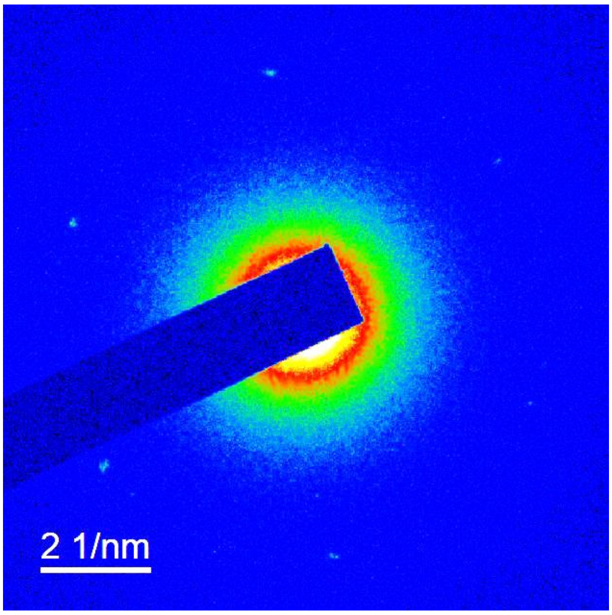

Supplementary Fig. 9 SAED pattern of a single RhMo NS.

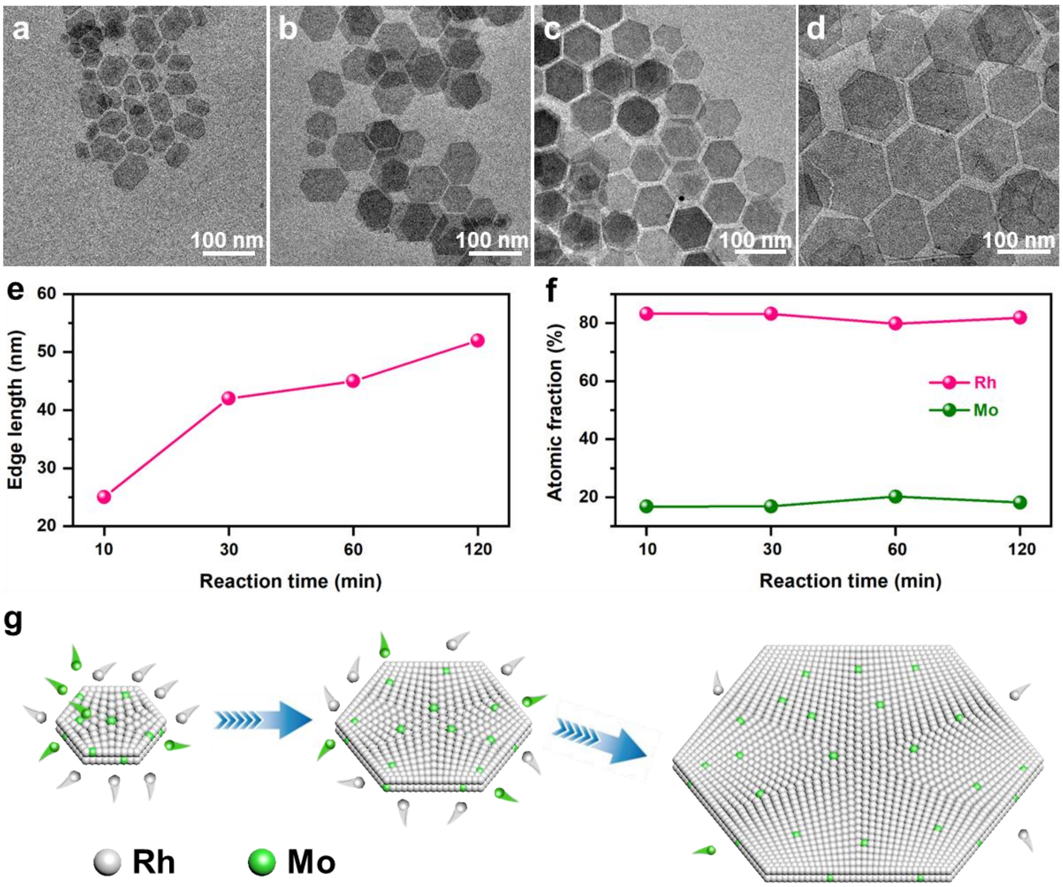

Supplementary Fig. 10. Growth mechanism of RhMo NSs. TEM images of intermediates obtained at (a) 10 min, (b) 30 min, (c) 60 min, and (d) 120 min. (e) Edge length and (f) SEM-EDS analysis of the intermediates. (g) Synthetic scheme for RhMo NSs.

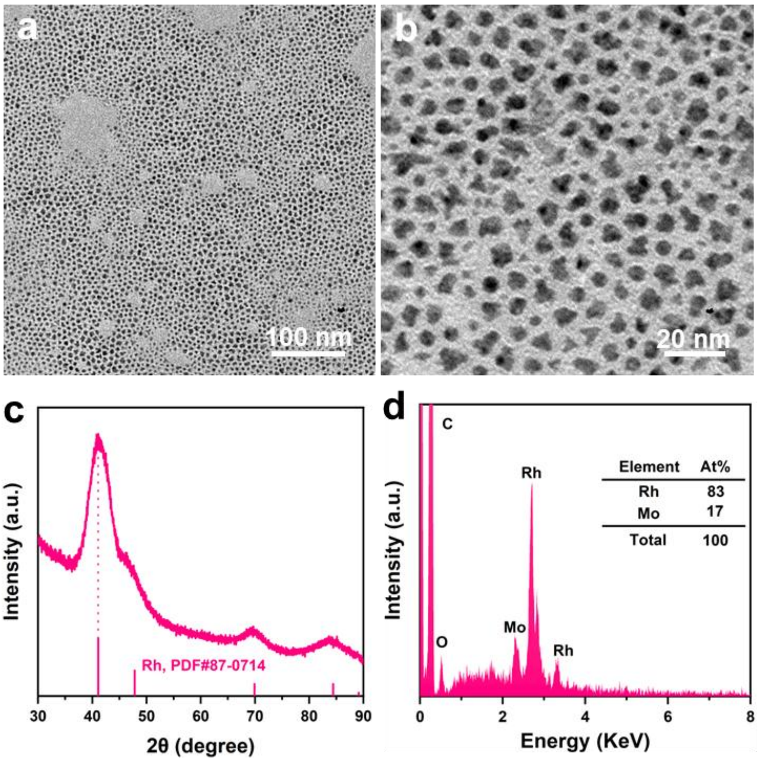

Supplementary Fig. 11 (a, b) TEM images, (c) XRD pattern, and (d) SEM-EDS analysis of the products synthesized by replacing $Mo(CO)_6$ with $MoCl_6$.

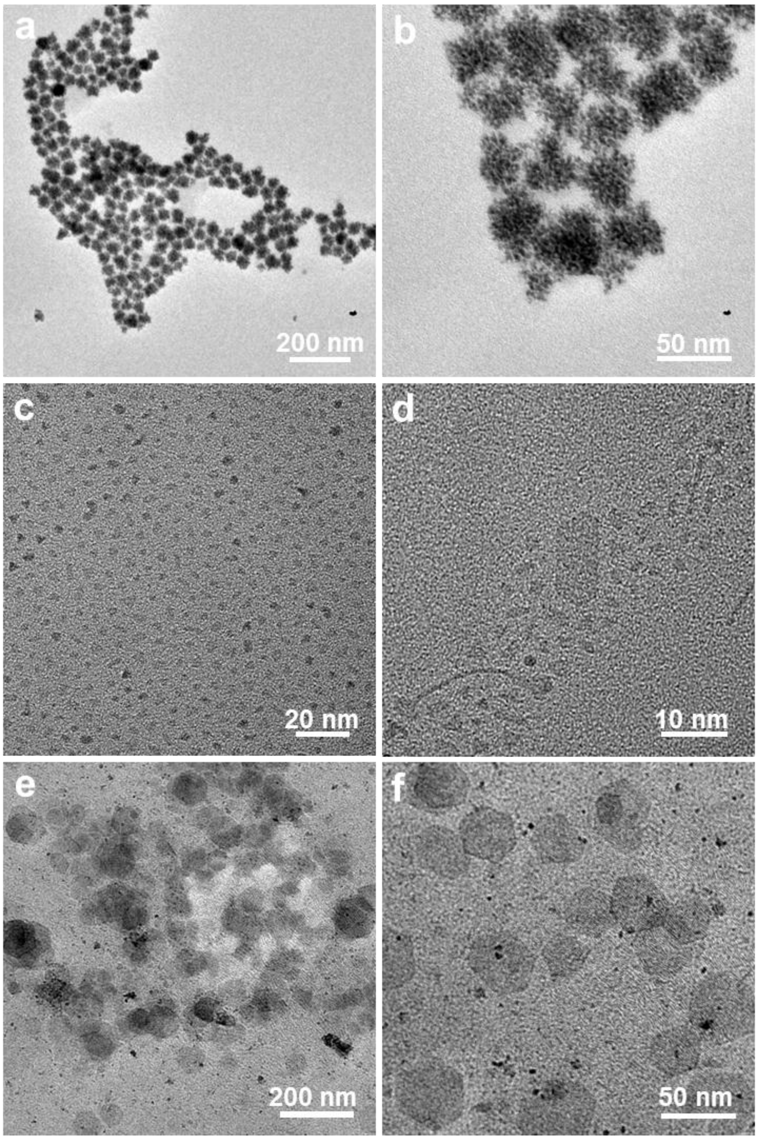

Supplementary Fig. 12 TEM images of the products synthesized with the same reaction conditions as those of RhMo NSs except the use of (a, b) 0 mg, (c, d) 10 mg, and (e, f) 100 mg $Mo(CO)_6$.

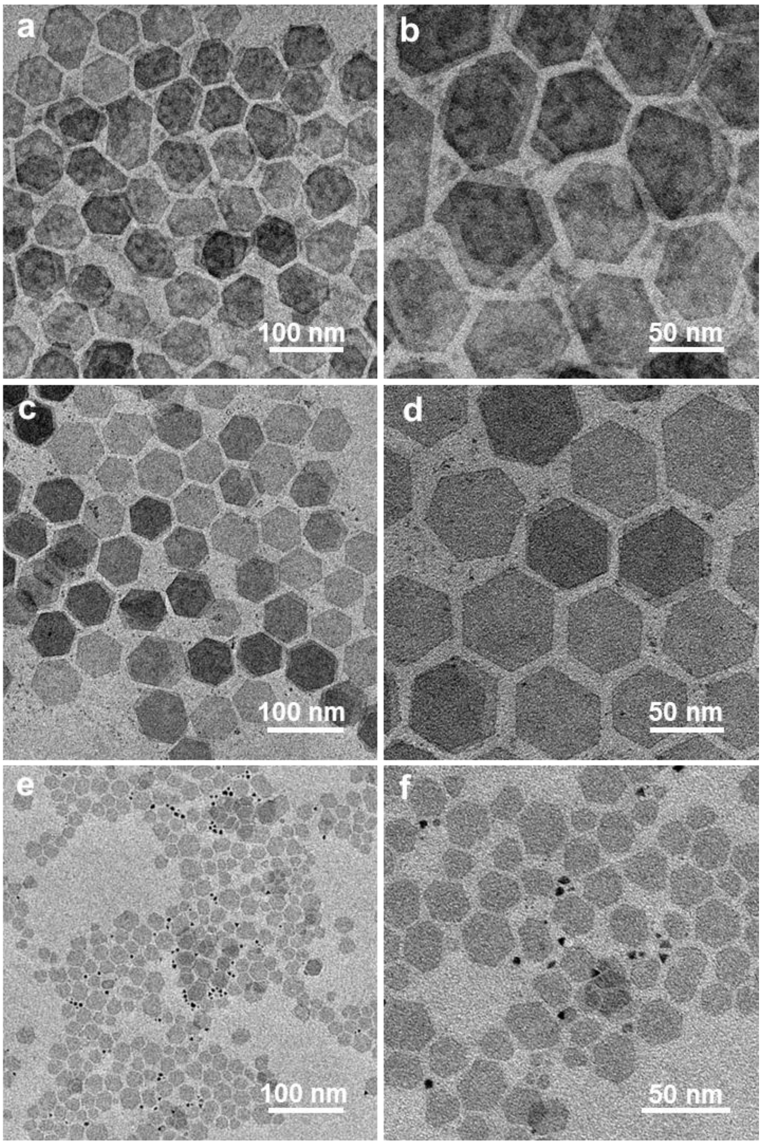

Supplementary Fig. 13 TEM images of the products with the same reaction conditions as those of RhMo NSs except the use of (a, b) 50 mg $Cr(CO)_6$, (c, d) 50 mg $W(CO)_6$, and (e, f) 50 mg $Fe_2(CO)_9$.

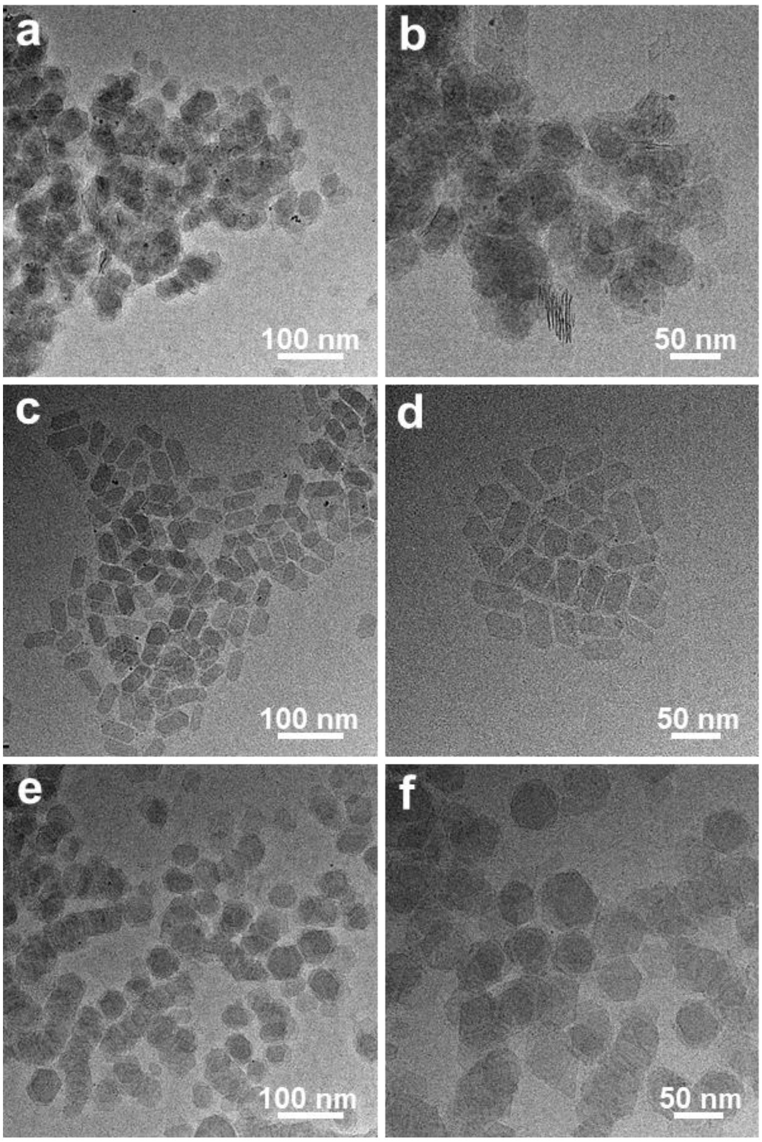

Supplementary Fig. 14 TEM images of products under the typical condition but varying the amounts of (a, b) 0 mg, (c, d) 16 mg, and (e, f) 64 mg KBr.

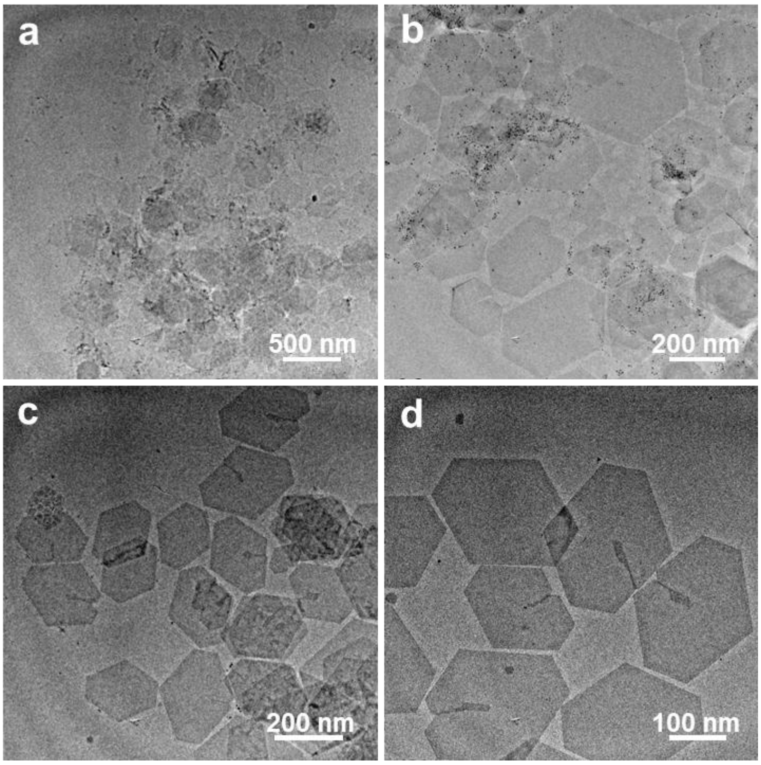

Supplementary Fig. 15 TEM images of the products synthesized with the same reaction conditions as those of RhMo NSs except replacing KBr with (a, b) CTAB, and (c, d) KCl.

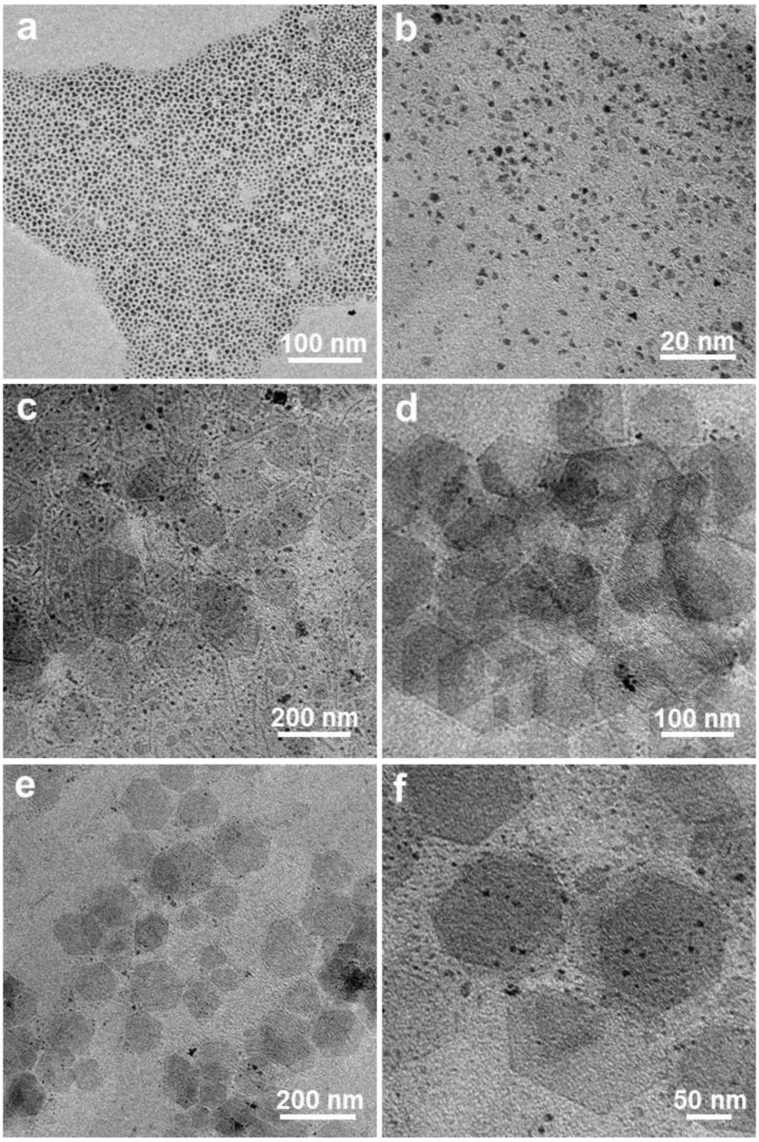

Supplementary Fig. 16 TEM images of the products synthesized with the same reaction conditions as those of RhMo NSs except the use of (a, b) 0 mg, (c, d) 50 mg, and (e, f) 200 mg CA.

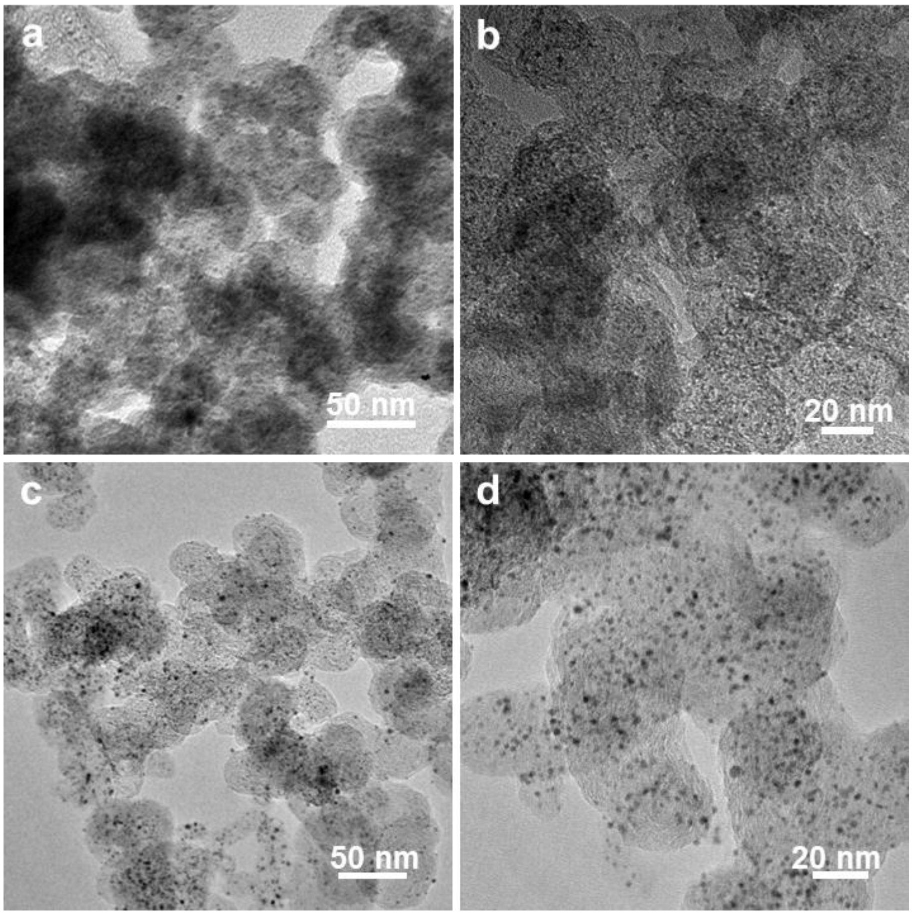

Supplementary Fig. 17 TEM images of (a, b) home-made Rh/C, and (c, d) commercial Pt/C.

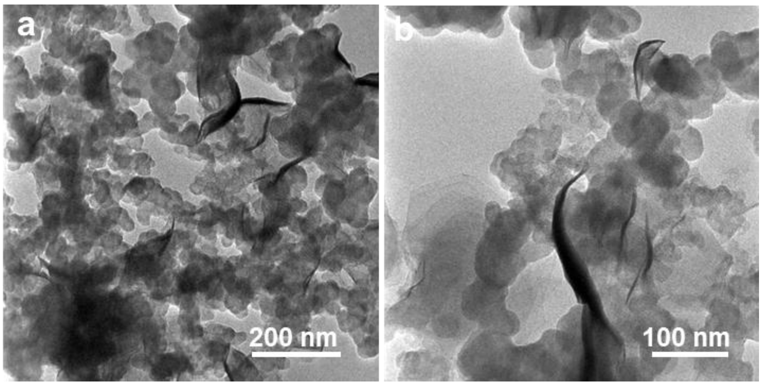

Supplementary Fig. 18 (a, b) TEM images of RhMo NSs/C.

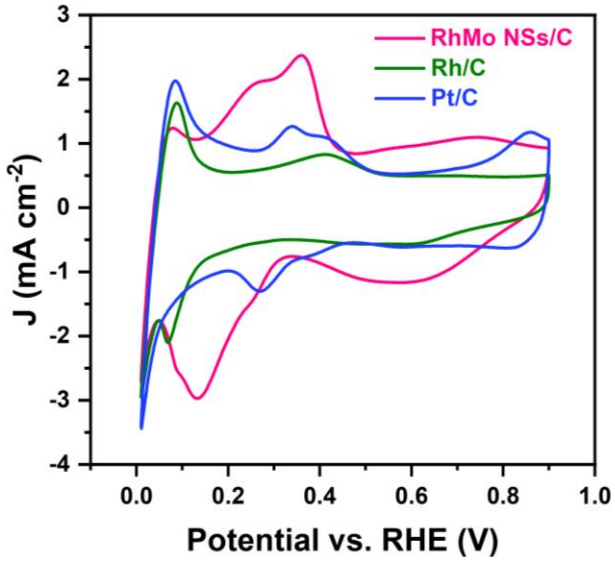

Supplementary Fig. 19 CV curves of RhMo NSs/C, Rh/C and Pt/C in N_2-saturated 0.1 M KOH at a scan rate of 100 mV s^{-1}.

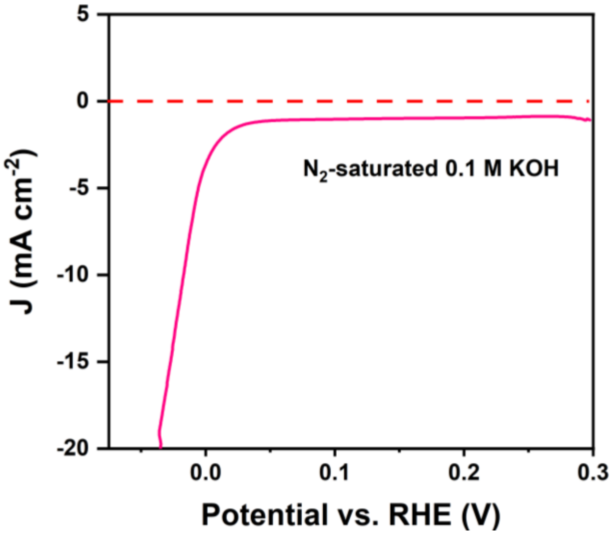

Supplementary Fig. 20 Polarization curve of RhMo NSs/C in N_2-saturated 0.1 M KOH.

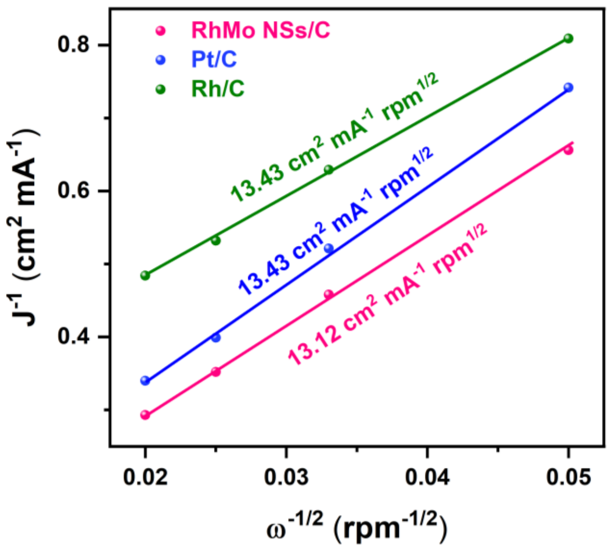

Supplementary Fig. 21 Koutecky-Levich plots of RhMo NSs/C, Rh/C and Pt/C at the overpotential of 100 mV.

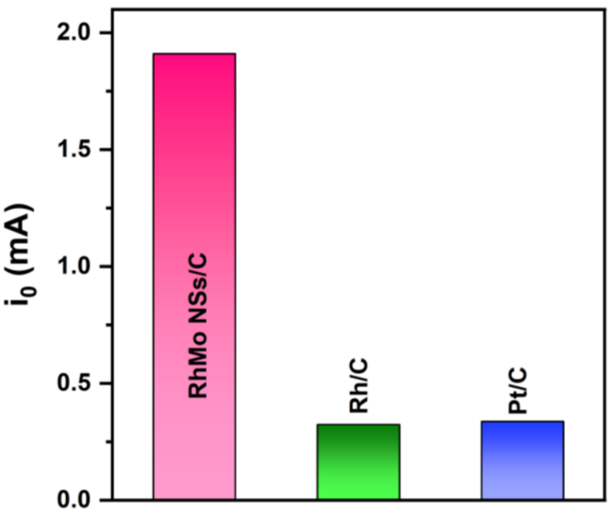

Supplementary Fig. 22 The exchange currents of RhMo NSs/C, Rh/C, and Pt/C.

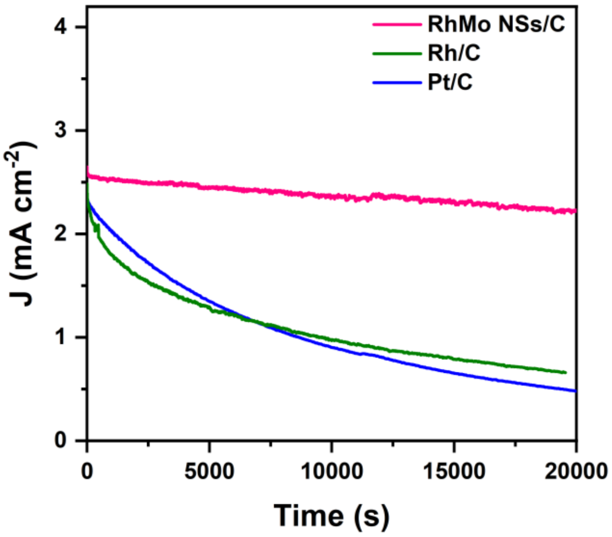

Supplementary Fig. 23 Relative current-time chronoamperometry responses of RhMo NSs/C, Rh/C and Pt/C in H_2-saturated 0.1 M KOH at the overpotential of 100 mV.

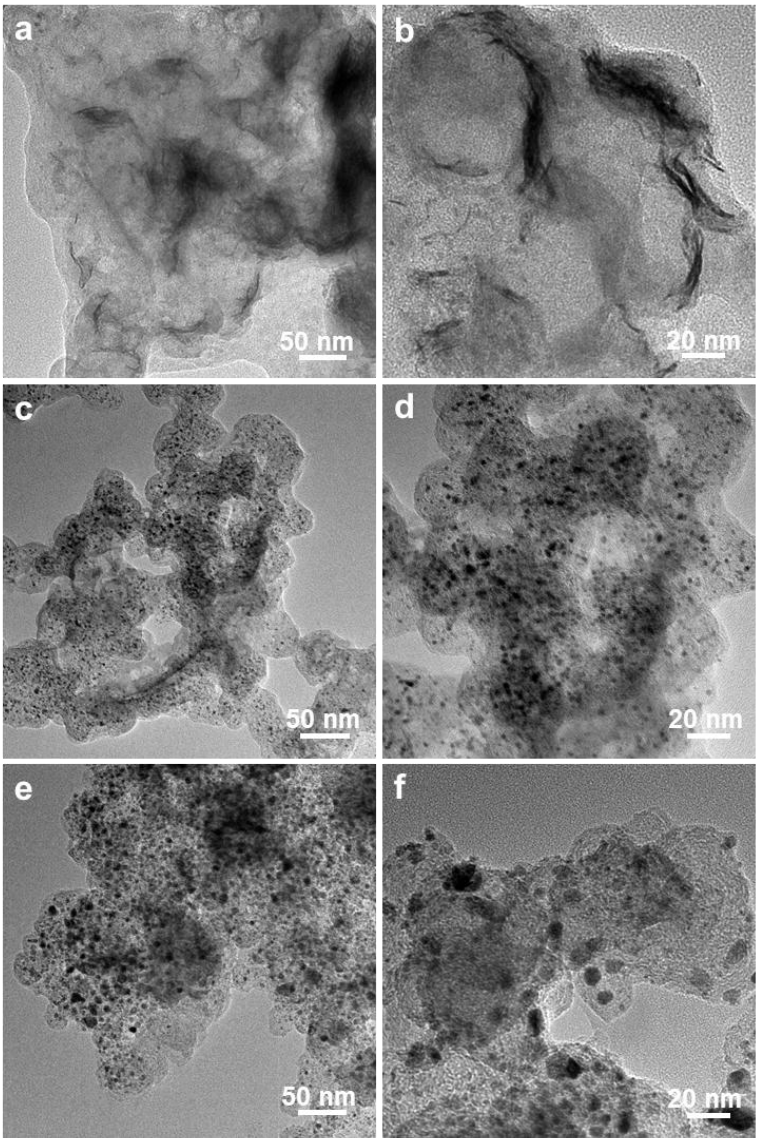

Supplementary Fig. 24 TEM images of (a, b) RhMo NSs/C, (c, d) Pt/C, and (e, f) Rh/C after HOR durability test.

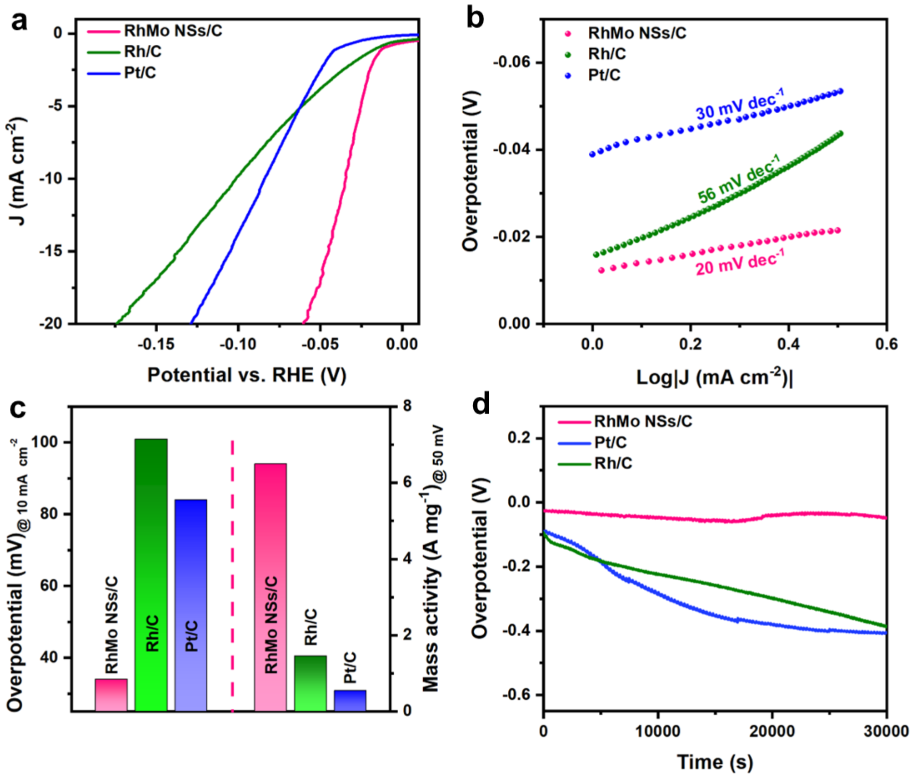

Supplementary Fig. 25 (a) HER LSV curves, and (b) Tafel slopes of RhMo NSs/C, Rh/C, and Pt/C. (c) Summary of overpotentials at the current density of 10 mA cm^{-2} and mass activity at the overpotential of 50 mV, and (d) 30000 s chronopotentiometry tests for RhMo NSs/C, Rh/C, and Pt/C.

a

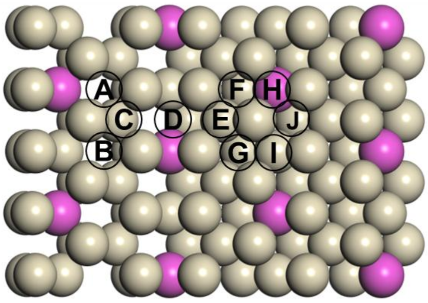

b

Site	ΔG(*H) (eV)	Site	ΔG(*H) (eV)
A	-0.26	F	-0.25
B	-0.08	G	-0.05
C	-0.22	H	-0.23
D	-0.30	I	-0.21
E	-0.10	J	-0.27

Supplementary Fig. 26 (a) Potential adsorption sites of hydrogen on fcc-hcp polymorph. (b) The corresponding hydrogen adsorption free energies.

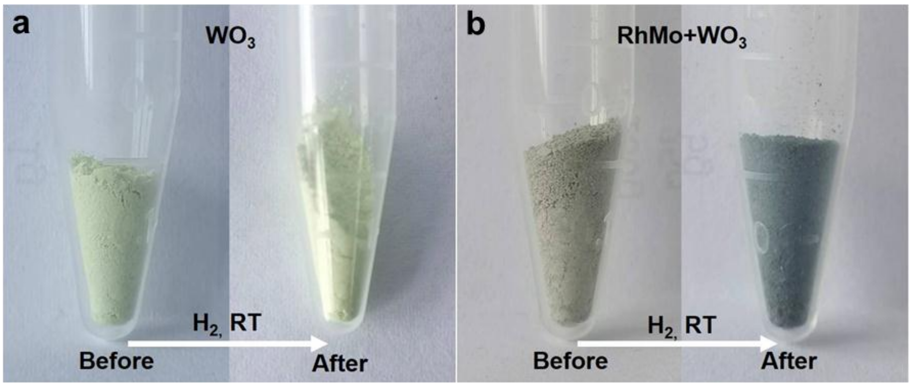

Supplementary Fig. 27 Photographic images of (a) WO_3 and (b) the mixture of RhMo NSs and WO_3 before and after H_2 treatment at room temperature.

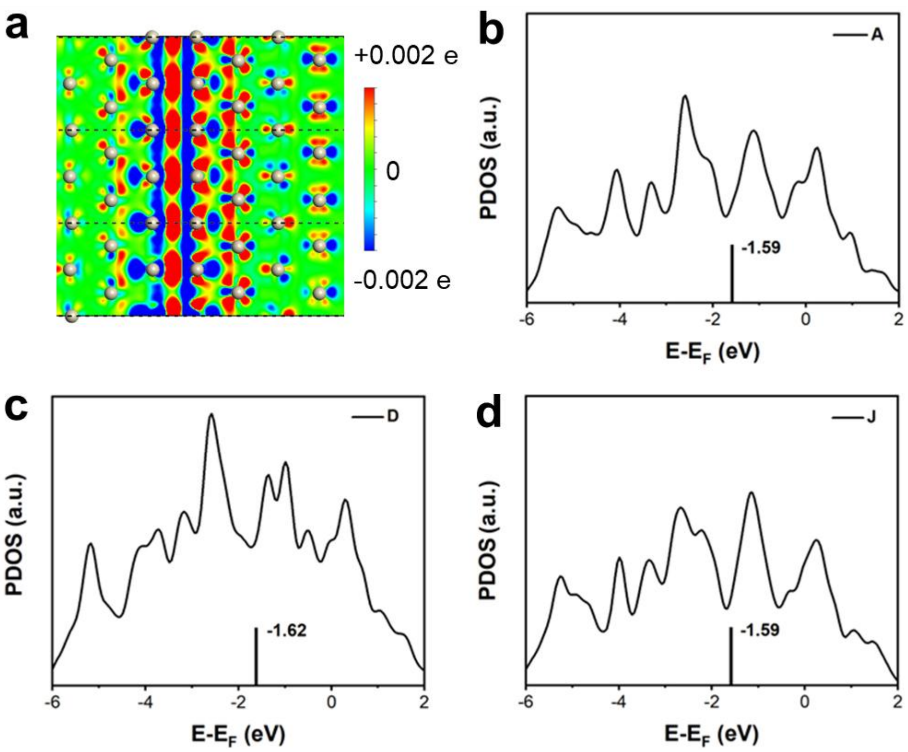

Supplementary Fig. 28 (a) Charge density difference plot of polymorphism RhMo structures and the projected d bands on site A, D and J to represent the electronic properties of (b) hcp (site A), (c) hcp-fcc (site D) and (d) fcc regions (site J).

Supplementary Table 1 Comparison of mass activity of RhMo NSs/C with other previous reported PGM catalysts for HOR in 0.1 M KOH.

**Catalyst**	**Mass activity (A mg_{PGM}^{-1})**	**Ref.**
**RhMo NSs**	**6.96**	**This work**
Rh_2Sb	3.56	*Adv. Mater.* **33**, 2105049 (2021).
Rh/C-2	0.68	*ACS Catal.* **9**, 5057-5062 (2019).
Rh_2P	0.52	*ChemElectroChem.* **6**, 1990-1995 (2019).
Rh NP/PC	0.16	*Small* **15**, 1903057 (2019).
Pt_6NCs/C	3.658	*Nat. Commun.* **13**, 1596 (2022).
Pt/NiO-300	2.86	*Nano Lett.* **21**, 4845-4852 (2021).
$Ru_{0.96}Pt_{0.04}$ NTs	2.58	*ACS Catal.* **5**, 7015-7023 (2015).
PtRu NWs	2.2	*ACS Catal.* **6**, 3895-3908 (2016).
$Pt_{0.1}Ru_{0.9}$	1.9	*Nat. Chem.* **5**, 300-306 (2013).
$Pt_{0.25}Ru_{0.75}$/N-C	1.654	*Adv. Mater. Interfaces* **7**, 2000310 (2020).
$Pt_{0.8}Ru_{0.2}$/C	0.696	*J. Phys. Chem. C* **119**, 13481-13487 (2015).
Pt/Cu NWs	0.65	*J. Am. Chem. Soc.* **135**, 13473-13478 (2013).
Acid-PtNi/C	0.474	*J. Am. Chem. Soc.* **139**, 5156-5163 (2017).
PtRu/Mo_2C-TaC	0.403	*ACS Catal.* **11**, 932-947 (2021).
PtNb/NbO_x-C	0.36	*ACS Catal.* **7**, 4936-4946 (2017).
PtRh	0.322	*J. Power Sources* **435**, 226798 (2019).
Pt/p-CNT	0.31	*Electrochim. Acta* **283**, 1829-1834 (2018).

Supplementary Table 2 Comparison of peak power density of HEMFC (H_2-O_2) operated with PGM-anode catalysts.

**Anode**	**Metal loading (mg_{PGM} cm^{-2})**	**Cathode (mg cm^{-2})**	**PPD (mW cm^{-2})**	**Ref.**
**RhMo NSs**	**0.2**	**0.2 (Pt/C)**	**1520**	**This work**
Pd-CeO_2/OLC	0.15	0.4 (Pt/C)	1000	*ACS Catal.* **12**, 7014-7029 (2022).
Pd-CeO_2	0.15	0.4 (Pt/C)	900	
Pd–CeO_2/C	0.2	0.4 (Pt/C)	1400	*ACS Appl. Energy. Mater.* **2**, 4999-5008 (2019).
IrNi@PdIr/C	0.1	0.3 (Pt/C)	311	*Nanoscale* **10**, 4872-4881 (2018).
$Pd_{0.33}Ir_{0.67}$/C	0.2	0.3 (Pt/C)	514	*J. Mater. Chem. A* **7**, 3161-3169 (2019).
Ru/C	0.5	0.5 (Pt/C)	250	*J. Power Sources* **225**, 311-315 (2013).
Pd-CeO_2/C	0.42	0.58 (Pt/C)	1000	*J. Electrochem. Soc.* **165**, J3039-J3044 (2018).
CeO_x-Pd/C	0.38	0.7 (Pt/C)	1169	*Adv. Funct. Mater.* **30**, 2002087 (2020).
PtRu/N-C	0.7	0.4 (Pt/C)	831	*Adv. Mater. Interfaces* **7**, 2000310 (2020).
Ru_7Ni_3/C	0.2	0.4 (Pt/C)	2030	*Nat. Commun.* **11**, 5651 (2020).
PtRu/C	0.6	0.4 (Pt/C)	2550	*Energy Environ Sci.* **12**, 1575-1579 (2019).
Ru/meso C	0.1	0.45 (Pt/C)	1020	*J. Power Sources* **461**, 2002087 (2020).
RuNi/NC	1	0.2 (Pt/C)	540	*Sci. Adv.* **8**, eabm3779 (2022).
PtRu/C	0.4	0.4 (Pt/C)	1890	*J. Power Sources* **166**, F3305-F3310 (2019).
PtRu/C	0.6	Ag/C	1100	*Chem. Commun.* **53**, 11771-11773 (2017).
Pd/C-CeO_2	0.3	Ag/C	500	*Angew. Chem. Int. Ed.* **55**, 6004-6007 (2016).
Pd/Ni	0.3	Ag/C	400	*J. Power Sources* **304**, 332-339 (2016).
PtRu/C	0.4	$CoMn_2O_4$/C	1100	*ACS Energy Lett.* **4**, 1251-1257 (2019).
Pt/C	0.4	Mn-Cospinel/C	1100	*Nat. Commun.* **10**, 1506 (2019).
Pd/CeO_2-C	0.25	Ag-Co	1000	*ACS Appl. Energy Mater.* **3**, 10209-12214 (**2020**).

PtRu/C	0.4	Fe/N/C	400	*ACS Catal.* **7**, 6458-6492 (2017).
PdIrRu/C	0.2	Ag/C	820	*Chem. Commun.* **56**, 5669-5672 (2020).
PtRu/C	0.9	$Fe_{0.5}$-NH_3	1040	*J. Electrochem. Soc.* **167**, 134505 (2020).
